# The Current Use of Drug-Eluting Balloons and Stents in Peripheral Arterial Disease: An Online Survey by the Cardiovascular and Interventional Radiological Society of Europe (CIRSE)

**DOI:** 10.1007/s00270-023-03562-3

**Published:** 2023-09-27

**Authors:** Robert A. Morgan, Stefan Müller-Hülsbeck, Fabrizio Fanelli, Patrick Haage, Mohamad Hamady, Romaric Loffroy, Gerard O’Sullivan, Florian Wolf, Birgit Slijepčević

**Affiliations:** 1 St George’s University of London, London, UK; 2 Academic Hospital Christian-Albrechts-University Kiel, Kiel, Germany; 3 Vascular and Interventional Radiology Department, “Careggi” University Hospital – University of Florence, Florence, Italy; 4 Helios University Hospital, University Witten/Herdecke, Wuppertal, Germany; 5 Imperial College, St Mary’s Campus, London, UK; 6 Department of Diagnostic and Interventional Radiology, University of Burgundy, Dijon, France; 7 University Hospital Galway, Galway, Ireland; 8 Division of Cardiovascular and Interventional Radiology, Medical University of Vienna, Vienna, Austria; 9 CIRSE Central Office, Vienna, Austria

**Keywords:** Drug-eluting devices, Current practice, Member survey, Paclitaxel-coated balloons, Paclitaxel-eluting stents

## Abstract

**Purpose:**

To assess the current use of drug-eluting devices for peripheral arterial disease (PAD) among interventional radiologists following the controversy caused by the 2018 meta-analysis suggesting an increased mortality risk for paclitaxel-eluting devices.

**Methods:**

An anonymous survey was sent to 7035 CIRSE members via email; only complete responses were included and statistically analysed.

**Results:**

Three hundred and seven members (4.4%) completed the survey. Among these, 95.8% indicated that they personally perform peripheral vascular procedures. Thirty-eight percentage of respondents did not see any change of practice since 2018, while 47% reported that the use of drug-eluting devices decreased; for 13%, the use stopped altogether, while it increased in 3% of responses. 45.6% of respondents also felt the impact of the controversy in terms of pricing, availability or directives from hospital administration. A large majority of respondents (83.7%) who perform peripheral vascular procedures consider the use of these devices as safe, 12.9% were undecided and 3.4% did not consider them as safe. Among the respondents who do not perform endovascular procedures, 77% considered these devices as safe and 23% were undecided.

**Conclusion:**

Although the 2018 meta-analysis had a disruptive impact on the use of drug-eluting devices in PAD, with the increasing body of evidence available, a majority of respondents continue to believe in the safety of these devices for use in femoropopliteal disease.

**Graphical Abstract:**

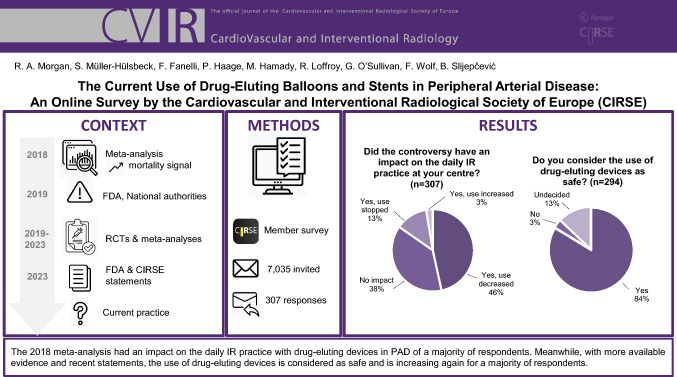

## Introduction

Since the meta-analysis of paclitaxel-eluting device use was published by Katsanos et al. in 2018 [1], the landscape on the use of drug-eluting devices for peripheral arterial disease (PAD) has undergone significant change. Although several studies have called into question the mortality signal raised in the paper since then, reactions to the meta-analysis by national authorities such as the US Food and Drug Administration (FDA) impacted daily IR practice in many regions. To better understand the real impact and current use of drug-eluting devices for PAD within the IR community more than 4 years after the controversy started, a CIRSE member survey was designed to better assess the effect of the article on PAD practice by IRs. In parallel, the society’s Endovascular Subcommittee (EVSC) worked on an Expert Opinion paper, concluding that the re-analysis of the current available data does not support a link between paclitaxel-eluting devices and mortality [2]. The analysis was published at the same time as an update letter to healthcare authorities by the FDA [3] on the mortality risk of paclitaxel-coated devices. The results of the CIRSE member survey are presented at a time when this topic is receiving significant attention, and we may start to see changes in practice again.

## Materials and Methods

A survey questionnaire was developed by the authors and programmed into an online survey tool (Alchemer). The anonymous survey was made available to 7035 CIRSE members on June 5, 2023. Two reminders were sent in the following weeks, and the survey was closed on July 6, 2023.

### Statistical Analysis

The collected data were analysed using statistical methodology, including primarily univariate and multivariate frequency analysis. All data analysis was performed in Microsoft Excel 2016.

## Results

A total of 307 complete responses were submitted, corresponding to a response rate of 4.4%. Among European CIRSE members who took the survey, the response rate amounted to 5.3%.

### General Demographics and Geographic Spread

With regard to the geographical distribution, a vast majority of respondents were European CIRSE members (80%). Members from Germany (17%), the United Kingdom (15%), Greece and Italy (6% each) and the Netherlands (5%) accounted for almost half of the responses. Among non-European members, Australian CIRSE members accounted for the biggest group with 4% of responses.

Respondents practice primarily at teaching or university hospitals (58%) or in general/public hospitals (31%); fewer respondents indicated that they work in private hospitals, clinics or foundations (10%).

Among the 307 respondents, 95.8% indicated that they personally perform peripheral vascular procedures.

### Impact of the Controversy on Drug-Eluting Devices

**Fig. 1:**
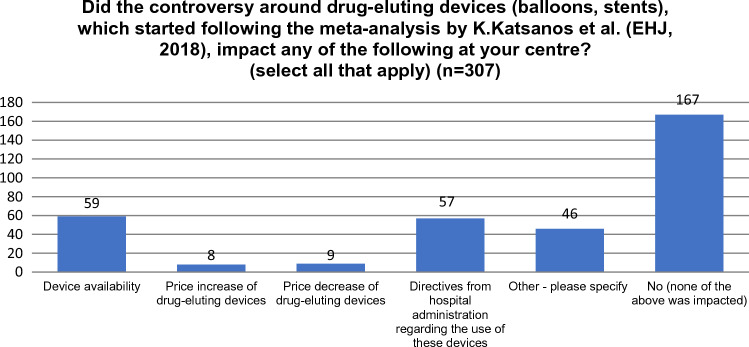
Impact of the controversy at the hospital level

Respondents were asked whether the controversy around drug-eluting devices (balloons, stents), which started following the meta-analysis by Katsanos et al. [1] impacted the availability, pricing or directives from hospital administration regarding the use of these devices at their centre (see Fig. 1). A majority of members (54.4%) responded that there was no impact on these areas at their centre. 19.2% indicated that there was an impact on device availability, while directives from hospital administration changed for 18.6%. Price increase (2.6%) or decrease (2.9%) was only reported by very few responders. Fifteen percent of respondents indicated that other aspects were impacted.

**Fig. 2:**
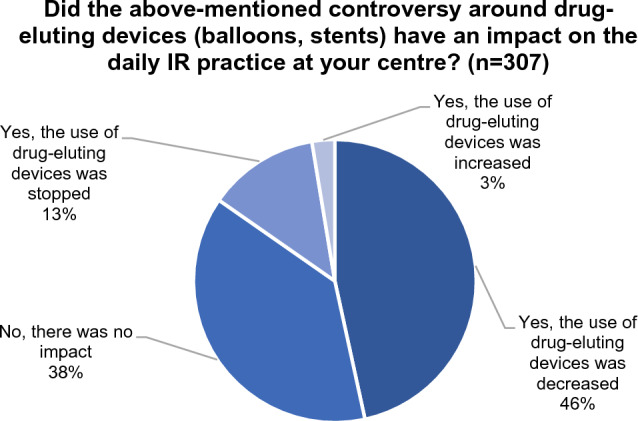
Impact of the controversy on daily IR practice

When asked about the impact of the meta-analysis on the daily IR practice at their centre, 38% of respondents did not see any change in practice, while 47% reported that the use of drug-eluting devices decreased. Thirteen percentage reported that the use of drug-eluting devices was stopped at their centre, while it increased in 3% of cases (see Fig. 2). Based on their response to this question, the sample was split into three subgroups who were then asked a different follow-up question.

All respondents who indicated that there was no impact on IR practice (*n* = 117) at their centre were asked if, meanwhile, IR practice has remained the same. In this group, 89.7% of respondents indicated that the use of drug-eluting devices was still the same at their centre as it was in 2019. The remaining 10.3% were asked to indicate what changed in an open response question; the most frequent response (open text) was that the use of these devices further increased.

Respondents who had replied that the use of drug-eluting devices increased (*n* = 8) or decreased (*n* = 143) due to the above-mentioned controversy were then asked whether their practice with drug-eluting balloons (DEBs) and drug-eluting stents (DESs) have remained the same since then. In this sub-sample, 48.3% of respondents indicated that their practice with drug-eluting devices has remained the same since then (including all who replied that it initially increased), while it changed for 52%. In the latter group (*n* = 78), 62.8% indicated in an open response explanation that the use of DEB/DES increased again, while 23 respondents (29.5%) indicated that practice further decreased.

If the use of DEB/DES had been stopped completely, respondents (*n* = 39) were asked whether the use of these devices had been taken up again in the meantime. Fifty-nine percentage of respondents in this subgroup have started using drug-eluting devices again. Forty-one percentage of respondents still do not use drug-eluting devices. The most frequent reasons (open text) for why it has not been taken up again include hospital directives and insufficient evidence or guidance.

### Safety of DEB/DES for PAD

**Fig. 3:**
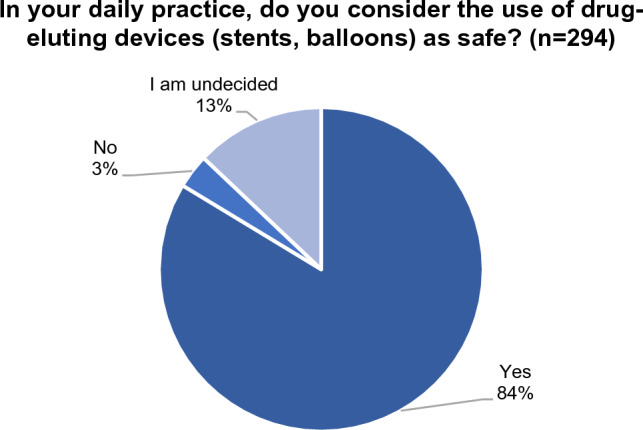
Opinion on safety of drug-eluting devices

All participants who indicated that they personally perform peripheral vascular procedures (95.8%) were asked whether they consider the use of drug-eluting devices as safe in their daily practice. An overwhelming majority of respondents (83.7%) replied positively, while 3.4% indicated that they do not consider the practice as safe. 12.9% were undecided (see Fig. 3).

IRs who do not personally perform peripheral vascular procedures (4.2%) were asked whether, from available literature and practice in their department, they consider the use of drug-eluting devices as safe. In this group, 77% replied positively, while 23% were undecided; no respondent replied negatively.

### Support for Endovascular Practice

In the last section of the questionnaire, CIRSE members were asked, which tools would help in their daily endovascular practice. The analysis of open responses showed four main clusters: firstly, the need for guidelines, standards and specific guidance documents in the field (15.3%); secondly the need for more (long-term) evidence on these therapies (7.2%); thirdly a request for an updated CIRSE statement (5.2%), which has been published in the meantime [2]; and fourthly authority approval and/or cancellation of warnings (2.9%). Finally, 80% of respondents were aware of the new European Certification for Endovascular Specialists (EBIR-ES), which aims to support IRs as experts in the endovascular field, and 15.3% were already certified.

## Discussion

The survey was answered predominantly by IRs who are active in the field (95.8%), and in 15.3% of cases by EBIR-ES holders. The sample was thus highly specialised and can be assumed to be knowledgeable about the Paclitaxel situation. While the CIRSE membership can be considered to be representative of interventional radiology practice in Europe, a potential selection bias must be acknowledged for the present survey, as IRs who work in the endovascular field were more likely to open and respond to this survey, as were those with strong opinions on the topic.

While the benefits of paclitaxel-device use in the femoropopliteal segment are supported by medium term outcomes data [2], more evidence on the benefits of drug-eluting stents in treatments below the knee, such as the long-term data on the PADI trial [4], are needed. The recent FDA statement concludes that the currently available data do not support an excess mortality risk [3]. Similarly, the CIRSE Statement [2] advocates the benefits of drug coated device use in the absence of a proven risk of mortality in patients with femoropopliteal disease.

The present survey illustrates the disruptive impact that one meta-analysis can have in a field that had until then been considered to be reasonably well supported by clinical data. In retrospect, it is surprising that a single publication could disrupt an entire field of medical practice to the extent that the 2018 Katsanos publication did. The world medical media reacted to the findings of this one publication by rapidly advertising the published potential mortality risk to all vascular practitioners. These headlines were assimilated by National Government agencies responsible for patient safety, and these agencies promoted cessation or severe restriction of Paclitaxel device use worldwide. Although this might be considered to be an overreaction, particularly in the light of the recent publications from the FDA and CIRSE, it was correct for governments and vascular specialists to be cautious because of the perceived mortality signal.

There are potential ways to prevent a similar situation from occurring again in future. As pointed out in several open responses in the survey, in a field that is largely based on industry-driven studies, evidence on long-term data was absent in all studies that evaluated Paclitaxel devices. Similarly deficient were independent studies with endpoints other than primary patency or target lesion revascularisation. In an evolving field such as peripheral arterial disease intervention, to avoid a similar situation with another novel device or device class, a more independent and open research culture should be adopted with longer patient follow-up and less focused endpoints.

## Conclusion

While recognising the importance of industry-driven research for the development and availability of medical devices globally, the authors make a plea for a more open research culture in interventional radiology. Independent scientific research with longer follow-up must be a fundamental component of endovascular therapies. This would be in the interests of the practitioners who know the benefits of these therapies, but ultimately in the interest of all patients who will benefit from well-researched and safe therapies and devices.
