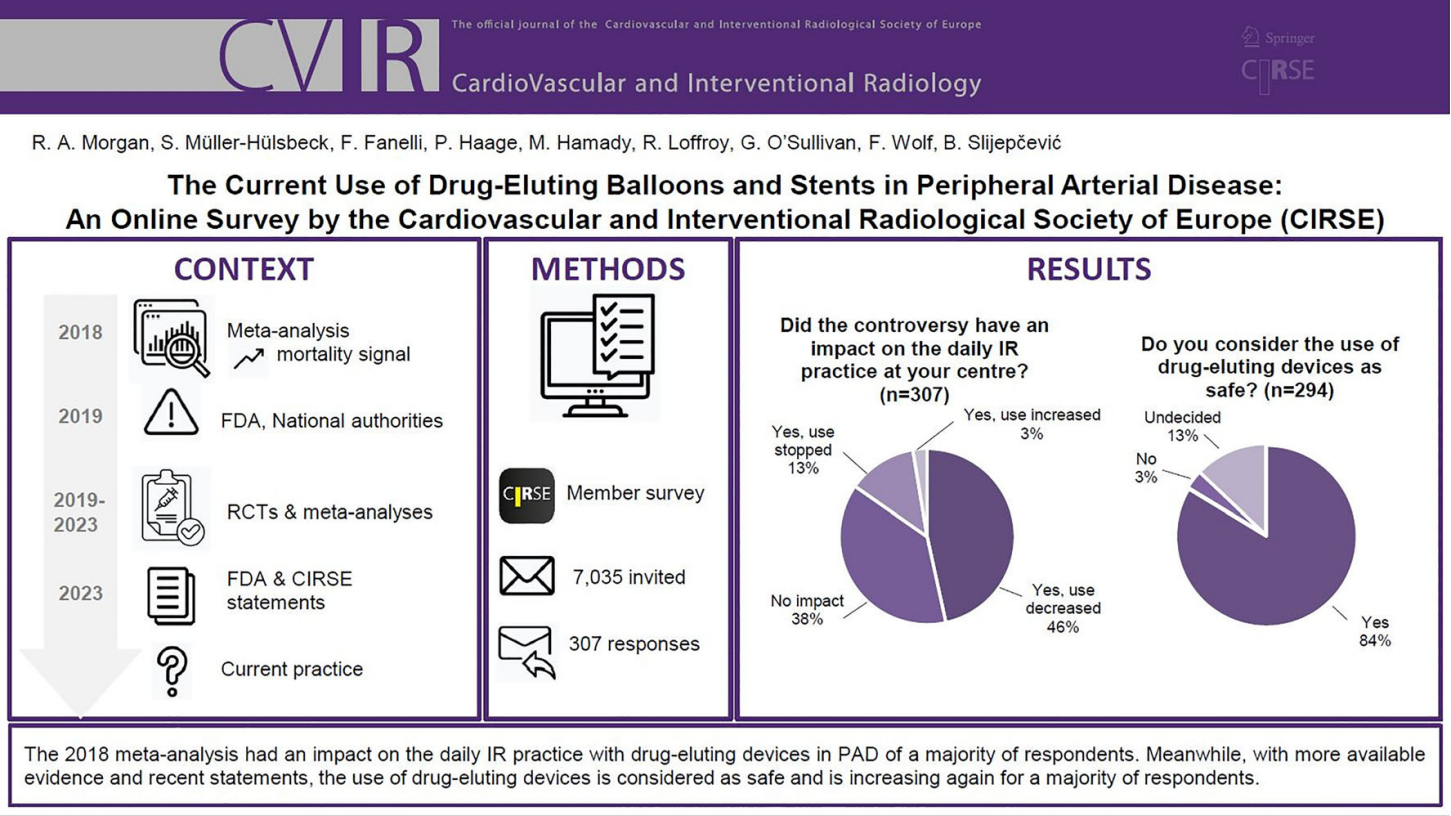# Correction to: The Current Use of Drug-Eluting Balloons and Stents in Peripheral Arterial Disease: An Online Survey by the Cardiovascular and Interventional Radiological Society of Europe (CIRSE)

**DOI:** 10.1007/s00270-023-03591-y

**Published:** 2023-11-01

**Authors:** Robert A. Morgan, Stefan Müller-Hülsbeck, Fabrizio Fanelli, Patrick Haage, Mohamad Hamady, Romaric Loffroy, Gerard O’Sullivan, Florian Wolf, Birgit Slijepčević

**Affiliations:** 1 St George’s University of London, London, UK; 2 Academic Hospital Christian-Albrechts-University Kiel, Kiel, Germany; 3 Vascular and Interventional Radiology Department, “Careggi” University Hospital – University of Florence, Florence, Italy; 4 Helios University Hospital, University Witten/Herdecke, Wuppertal, Germany; 5 Imperial College, St Mary’s Campus, London, UK; 6 Department of Diagnostic and Interventional Radiology, University of Burgundy, Dijon, France; 7 University Hospital Galway, Galway, Ireland; 8 Division of Cardiovascular and Interventional Radiology, Medical University of Vienna, Vienna, Austria; 9 CIRSE Central Office, Vienna, Austria

**Correction to: Cardiovasc Intervent Radiol** 10.1007/s00270-023-03562-3

The original article was published without the following visual abstract and has been corrected: